# Functional Outcomes and Their Influencing Factors for the Surgical Management of T-type Acetabulum Fractures: A Prospective Single-Centre Study of 73 Patients

**DOI:** 10.7759/cureus.59170

**Published:** 2024-04-27

**Authors:** Sandeep Kumar, Vivek Bhambhu, Rohit Goyal, Shataayu Gugale, Ganpat Choudhary, Akash Mishra, Akshay Yadav, Naresh Porwal

**Affiliations:** 1 Orthopaedics, Mahatma Gandhi Medical College and Hospital, Jaipur, Jaipur, IND; 2 Biostatistics, Mahatma Gandhi Medical College and Hospital, Jaipur, Jaipur, IND; 3 Orthopaedics, Keshav Porwal Hospital, Bhilwara, IND

**Keywords:** t with posterior wall fractures, t type fractures, functional outcomes, surgical approach, acetabulum fractures

## Abstract

Introduction

T-type fractures of the acetabulum are uncommon injuries, typically resulting in poorer long-term outcomes compared to other patterns of acetabular fractures. Our main purpose is to analyse the epidemiology, functional outcomes, and factors affecting the functional outcomes of patients with T-type acetabular fractures.

Methods

This prospective, single-centre study included 73 patients with T-type and T with posterior wall acetabular fractures. They underwent treatment with open reduction internal fixation using plating through the modified Stoppa, Kocher-Langenbeck (KL), or dual approach. The post-operative reduction was assessed according to Matta’s criteria, and functional outcomes were evaluated using the modified Harris hip score.

Results

Between September 2017 and January 2023, 53 patients underwent surgery for T-type fractures (72.6%), and 20 patients were treated for T with posterior wall acetabular fractures (27.4%). The minimum follow-up period was one year, with a mean follow-up of 3.5 years. Anatomical reduction emerged as the major contributing factor towards good functional outcomes compared to satisfactory reduction according to Matta’s criteria (P value: 0.006). Overall, 65 patients (89%) achieved excellent to good modified Harris hip scores, while eight patients (11%) obtained fair to poor scores. Patients with T-type fractures demonstrated better functional outcomes compared to T with posterior wall fractures (P value: 0.031).

Conclusion

Anatomical reduction, as assessed by Matta’s reduction criteria, serves as a predictor of favourable functional outcomes. T with posterior wall fractures exhibit poor outcomes in comparison to T-type fractures. The surgical approach employed does not influence the reduction or the final functional outcome of the patient.

## Introduction

T-type fractures of the acetabulum are uncommon injuries, representing 10%-40% of all acetabular fractures [1,2]. They pose challenges in treatment, particularly when associated with fractures of the posterior wall or a caudally oriented T-component [2-4]. These fractures typically result in poorer long-term outcomes compared to other patterns of acetabular fractures [3,5-7], with the complex regional anatomy and presence of a posterior wall fracture being among the main causative factors.

The choice of surgical approach remains a topic of debate. Currently, the Arbeitsgemeinschaft für Osteosynthesefragen (AO) Foundation recommends an anterior/posterior approach for displaced T-type fractures [2]. However, there is no universal consensus regarding the preference for any single approach for a specific fracture type. The conventionally used extensile approach is linked with complications such as heterotopic ossification, infections, post-traumatic arthritis, avascular necrosis [2], and loss of abductor power [5], making it challenging to achieve an optimal outcome [2].

The literature indicates outcome variations with various risk factors, including age, comorbidities, other associated injuries, and initial fracture displacement, all of which significantly alter the course of treatment [2]. Factors such as dome impaction, residual gaps and steps [3], delay in surgery, and choice of approach are also important determinants affecting outcomes [3].

We aim to analyse the epidemiology, outcomes, and factors influencing outcomes for patients with T-type acetabular fractures. To the best of our knowledge, only a limited number of clinical studies focusing specifically on this subject have been published to date [1-7].

## Materials and methods

Patients

A total of 538 acetabular fractures were presented to our institution between September 2017 and January 2023 and were initially managed according to Advanced Trauma Life Support (ATLS) protocols [8]. It is a prospective study, and consent was obtained from each subject regarding the use of their data for this study’s publication. Approval was obtained from the Institutional Research Ethics Committee of Mahatma Gandhi Medical College and Hospital, Jaipur, India (approval no. MGMCH/IEC/JPR/2017/110). No external source of funding was used for this study. T-type fractures with or without involvement of the posterior wall and patients who gave consent for this study were eligible for inclusion. A total of 75 patients were identified, out of whom two were excluded: one patient had a floating hip and the other operated on three weeks after the injury. Other elementary/associated types of fractures were excluded. Based on the above criteria, data were collected from 73 patients, including demographics, laboratory investigations, preoperative radiographs (anteroposterior, iliac, and obturator views) and computed tomography (CT) scans from previous reports and in-patient records. Patients were followed up for one to six years, with a mean follow-up of 32.03 months.

Procedure

Operative intervention was performed by a single surgeon trained in managing pelvi-acetabular injuries, with the approach chosen based on the fracture configuration. All patients underwent surgery on a standard operating table in either a supine or lateral position. Intraoperatively, less comminuted columns were addressed and used as pillars for the reduction of other columns. The implants used included stainless steel pelvic curved, infrapectineal, J-plates (R-88), spring, straight, suprapectineal, symphyseal, and dynamic compression (DC) hole reconstruction curved plates (Nebula Surgicals), which were fixed with cortical and cancellous screws as indicated. Fluoroscopic images of the pelvis with both hips (anteroposterior views and iliac and obturator views) were taken to assess the quality of reduction. 

Post-operative X-rays were conducted to assess reduction and classified according to Matta’s criteria [6]. A post-operative gap or step of <1 mm was categorised as anatomical reduction, 1-3 mm as imperfect reduction, and >3 mm as poor reduction. The range of motion exercises and in-bed mobilisation was initiated on post-operative day one. Drain removal was performed on day two. Weight-bearing was typically initiated at an average of 2.5 months after the operative procedure.

Statistical analysis

All categorical variables were categorised into modifiable and non-modifiable factors. Non-modifiable factors included age, gender, mode of injury, type of fracture, displacement category, transverse limb, vertical limb, marginal head impaction, and protrusion dislocation. The surgical approach used, posterior column (PC) plating with modified Stoppa, Matta's reduction criteria, and surgical delay were considered modifiable factors. These variables, along with the modified Harris hip score were summarised as frequency with percentage. The associations between various clinical variables were examined specifically, the associations of functional outcome with other clinical variables, age with displacement categories, type of fracture and Matta's reduction criteria with the approach used, age with type of fracture, mode of injury with protrusion dislocation, and mode of injury with marginal head impaction, using the chi-square test/Fisher's exact test. All the analyses were conducted at a 5% level of significance, and a P value < 0.05 was considered statistically significant.

## Results

Out of 73 patients, 53 (72.6%) were males, and the remaining 20 (27.4%) were females (Table 1). The ages of the patients ranged between 18 and 80 years and were divided into two groups: less than 40 years (Group A) and more than 40 years (Group B) to evaluate the effect of age on outcomes. Group A comprised 35 patients (47.9%), while Group B included 38 patients (52.1%), with a mean age of 40.24 years. In Group A, 24 patients (68.6%) had less than 30 mm initial displacement, while 11 patients (31.4%) had greater than 30 mm initial displacement. In Group B patients, 13 patients (34.2%) had less than 30 mm displacement, and 25 patients (65.8%) had greater than 30 mm initial displacement (P value: 0.003) (Table 1). Regarding the mode of injury, 50 patients had a history of motor vehicle collision (68.5%), and 23 patients fell from height (31.5%) (Table 1).

**Table 1:** Clinical characteristics of patients RTA: Road traffic accident; FFH: Fall from height

Characteristics	n (%)
Non-modifiable factors	
Age (years)	
< 40	35 (47.9)
≥ 40	38 (52.1)
Gender	
Male	53 (72.6)
Female	20 (27.4)
Mode of injury	
RTA	50 (68.5)
FFH	23 (31.5)
Type of fracture	
T-type	53 (72.6)
T-type with posterior wall	20 (27.4)
Displacement category	
< 30	37 (50.7)
≥ 30	36 (49.3)
Transverse limb	
Juxtatectal	31 (42.5
Transtectal	42 (57.5)
Vertical limb	
Posterior	12 (16.4)
Vertical	53 (72.6)
Anterior	8 (11.0)
Marginal head impaction	
Yes	14 (19.2)
No	59 (80.8)
Protrusion dislocation	
Yes	44 (60.3)
No	29 (39.7)
Modifiable factors	
Approach used	
Modified Stoppa	33 (45.2)
Kocher-Langenbeck	28 (38.4)
Dual	12 (16.4)
Matta's reduction criteria	
< 1 mm	34 (46.6)
1-3 mm	39 (53.4)
Surgical delay	
< 7 days	64 (87.7)
≥ 7 days to < 3 weeks	9 (12.3)
Functional outcome measure	
Modified Harris hip score	
Excellent and good	65 (89.0)
Poor and fair	8 (11.0)

We operated on a total of 73 patients, including those with T-type fractures (53, 72.6%) and T-type with posterior wall fractures (20, 27.4%). Fracture patterns were subdivided based on transverse and vertical fracture lines. The transverse limb was divided into transtectal (42 patients), juxtatectal (31 patients), and infractectal (0 patients), while the vertical limb was divided into posterior, vertical, and anterior parts with 12, 53, and eight patients, respectively (Table 1).

In patients with T-type fractures, 33 patients (62.3%) were addressed through the modified Stoppa approach, 14 patients (26.4%) through the Kocher-Langenbeck approach (KL), and the remaining six patients (11.3%) through the dual approach. Conversely, among patients with T-type fractures with posterior wall involvement, 14 (70%) were operated on using the KL approach, and the remaining six (30%) through the dual approach (P value: <0.001) (Table 2). Patients operated on within seven days of injury exhibited excellent to good outcomes in 59 cases and fair to poor outcomes in five cases. Among patients operated on after seven days, six patients had excellent to good functional outcomes, while three patients had fair to poor outcomes (P value: 0.054) (Table 2).

**Table 2:** Association of functional outcomes with other variables RTA: Road traffic accident; FFH: Fall from height

Variables		Modified Harris hip score		P value
		Excellent and good (n = 65)	Poor and fair (n = 8)	
		n (%)	n (%)	
Non-modifiable factors				
Age (years)	< 40	32 (91.4)	3 (8.6)	0.801
	≥ 40	33 (86.8)	5 (13.2)	
Mode of injury	RTA	43 (86.0)	7 (14.0)	0.211
	FFH	22 (95.7)	1 (4.3)	
Type of fracture	T-type	50 (94.3)	3 (5.7)	0.031
	T-type with posterior wall	15 (75.0)	5 (25.0)	
Displacement categories	< 30	33 (89.2%)	4 (10.8%)	1.00
	≥ 30	32 (88.9%)	4 (11.1%)	
Transverse limb	Juxtatectal	29 (93.5)	2 (6.5)	0.454
	Transtectal	36 (85.7)	6 (14.3)	
Vertical limb	Posterior	12 (100.0)	0 (0.0)	0.310
	Vertical	45 (84.9)	8 (15.1)	
	Anterior	8 (100.0)	0 (0.0)	
Marginal head impaction	Yes	13 (92.9)	1 (7.1)	1.000
	No	52 (88.1)	7 (11.9)	
Modifiable factors				
Approach used	Modified Stoppa	31 (93.9)	2 (6.1)	0.364
	Kocher-Langenbeck	23 (82.1)	5 (17.9)	
	Dual	11 (91.7)	1 (8.3)	
Matta's reduction criteria	< 1 mm	34 (100.0)	0 (0.0)	0.006
	1-3 mm	31 (79.5)	8 (20.5)	
Surgical delay	< 7 days	59 (92.2)	5 (7.8)	0.054
	≥ 7 days to < 3 weeks	6 (66.7)	3 (33.3)	

According to Matta’s criteria, 34 (46.6%) patients achieved anatomical reduction (<1 mm), while 39 patients had imperfect reduction (1-3 mm). All 34 patients who achieved anatomical reduction post-operatively had excellent to good functional outcomes. Among the remaining 39 patients with imperfect reduction, 31 (79.5%) patients experienced excellent to good functional outcomes, while eight (20.5%) patients had fair to poor functional outcomes (P value: 0.006) (Table 2).

Among the isolated T-type fractures, 50 patients (94%) achieved excellent to good functional outcomes, while three patients (5.7%) had fair to poor outcomes. In T-type fractures with posterior wall involvement, 15 patients (75%) experienced excellent to good outcomes, while five patients (25%) had fair to poor outcomes (P value: 0.031) (Table 2).

Out of the 73 patients, two presented with sciatic nerve palsy (Medical Research Council (MRC) grade: 0/5). Upon final assessment, one patient achieved complete recovery (MRC grade: 5/5), and the other patient experienced partial recovery (MRC grade: 3/5). In operative patients, two individuals developed early superficial surgical site infections, which were successfully managed by culture-specific antibiotics (intravenous + oral).

## Discussion

T-type fractures are reported to have an incidence of 10%-40% when considering overall elementary and associated fracture patterns of the acetabulum [1]. However, our study reported an incidence of 13.94% (75/538). The mean age of presentation in our study was 40.24 years, indicating a prevalence of these fractures in a younger population.

When variables related to fracture patterns are studied, only the type of fracture is significantly associated with the functional outcomes of the patient (P value: 0.031) (Table 2). In our study, 50 patients (94.3%) with T-type fractures had excellent to good outcomes as compared to 15 patients (75%) with T-type fractures involving the posterior wall. Yang et al. operated on 21 patients with T-shaped acetabular fractures involving the posterior wall and found excellent to good clinical outcomes in nine patients [4]. Similarly, Hammad et al. studied 34 patients and reported 25 patients with excellent to good outcomes [5]. However, components of the transverse or vertical limb do not affect the functional outcome. Other non-modifiable risk factors, such as marginal impaction and impaction of the femoral head and posterior dislocation or protrusion of the femoral head, do not have any significant association with the final functional outcomes of the patient (Table 2). This contrasts with the study by Jang et al. [3] where the juxtatectal/transtectal component showed a significant association with post-operative osteoarthritis, directly affecting the functional outcome of the patient.

The most common mechanism of injury was motor vehicle collisions (MVC) in 50 patients (68.5%), followed by falls from height (31.5%). A study by Kim et al. reported MVC in 59.1% of patients [2], while Yang et al. found MVC in 61.9% of their patients [4]. However, this mechanism of injury does not affect the final functional outcome of the patient (P value: 0.211). Additionally, the mode of injury also does not have a significant impact on posterior femoral head dislocation, marginal head impaction, or the initial displacement of fractures.

There is a lack of consensus on the optimal approach for different patterns of T-type fractures and their influence on clinico-radiological outcomes. Yang et al. stated that selecting the optimal surgical approach for these fractures is based on addressing the larger displacement and higher level of the T-type fracture, with the KL approach being used in obvious posterior displacement of most T-type fractures [4,9-13]. Kim et al. similarly used the KL approach in 61% of their patients, the extended iliofemoral approach in 19%, and the ilioinguinal approach in 13% [2]. Gusic et al. [14] recommend the KL approach in all T-type fractures except those associated with anterior wall involvement. In our study, we predominantly used the modified Stoppa approach in 33 patients (45.2%) and the KL approach in 28 patients (38.4%), while a dual approach (modified Stoppa + KL) was used in 12 patients (16.4%). However, the choice of approach did not have any significant effect on the post-operative functional outcome of the patients (P value: 0.364) (Table 2). Additionally, we did not find any statistically significant association between the approach used and post-operative reduction according to Matta’s criteria (P value: 0.443) (Table 2).

Intraoperative reduction of displaced columns is a modifiable factor. In our study, anatomically reduced fractures (34 patients) are associated with better functional outcomes than imperfectly reduced fractures (39 patients) (P value: 0.006). Matta et al. [6] operated on 31 T-shaped acetabular fractures and achieved anatomical reduction in 16 patients (52%), imperfect reduction in 10 patients (32%), and poor reduction in five patients (16%), further reporting excellent outcomes in six (19%), good in 18 (58%), fair in two (6%), and poor in five (16%) cases. Conversely, Yang et al. [4] stated anatomical and imperfect reductions in 12 patients (57.1%) and reported excellent to good outcomes in 42.9% of cases. Frietman et al. [15], in their study of 220 patients with acetabular fractures, similarly identified unsatisfactory reduction as a risk factor for poor outcomes, while Verbeek et al. [16], who operated on 227 patients with acetabular fractures, identified a post-operative gap of greater than or equal to 5 mm as a risk factor for poor outcomes.

Patients who were operated on within seven days of injury exhibited better functional outcomes as compared to those with a delay of more than seven days. Among the patients operated within seven days, 59 patients (92.2%) had excellent to good functional outcomes. A surgical delay of more than or equal to seven days was associated with excellent to good outcomes in only six (66.7%) patients and fair to poor outcomes in three (33.3%) patients (Table 2). Kim et al. [2] similarly reported better reductions and clinical outcomes with early fixation (<14 days post-injury). Meena et al. [17] operated on 118 patients and identified a surgical delay of more than 14 days as a risk factor for poor outcomes.

The limiting factor of our study is the need for longer follow-up periods to assess long-term outcomes and potential complications associated with T-type acetabulum fractures and their treatments.

## Conclusions

Posterior wall involvement, surgical delay more than seven days, and post-operative residual gaps and steps are the most important predictors that affect the functional outcomes. Other factors such as age, initial displacement of fractures, femoral head dislocation and protrusion, marginal impaction, and surgical approach, do not significantly affect the functional outcomes.

The risk factors for poor outcomes identified in our study exhibit some similarities with those identified in other studies, highlighting the need for further in-depth research to analyse these distinct factors.
